# Natural antibiotics against antimicrobial resistance: sources and bioinspired delivery systems

**DOI:** 10.1007/s42770-024-01410-1

**Published:** 2024-06-18

**Authors:** Beatriz N. Guedes, Karolline Krambeck, Alessandra Durazzo, Massimo Lucarini, Antonello Santini, M. Beatriz P. P. Oliveira, Faezeh Fathi, Eliana B. Souto

**Affiliations:** 1 Laboratory of Pharmaceutical Technology, Faculty of Pharmacy, University of Porto, Porto, 4050-313 Portugal; 2 Health Sciences School, Guarda Polytechnic Institute, Rua da Cadeia, Guarda, 6300-035 Portugal; 3 CREA-Research Centre for Food and Nutrition, Via Ardeatina 546, Rome, 00178 Italy; 4 Department of Pharmacy, University of Napoli Federico II, Via D. Montesano 49, Napoli, 80131 Italy; 5 REQUIMTE/LAQV, Department of Chemical Sciences, Faculty of Pharmacy, University of Porto, Rua Jorge Viterbo Ferreira, 280, Porto, 4050-313 Portugal

**Keywords:** Antimicrobial resistance, Natural antibiotics, Plant-based antibiotics, Algae-based antibiotics, Bioinspired delivery systems

## Abstract

The current burden associated to multidrug resistance, and the emerging superbugs, result in a decreased and even loss of antibiotic efficacy, which poses significant challenges in the treatment of infectious diseases. This situation has created a high demand for the discovery of novel antibiotics that are both effective and safe. However, while antibiotics play a crucial role in preventing and treating diseases, they are also associated with adverse effects. The emergence of multidrug-resistant and the extensive appearance of drug-resistant microorganisms, has become one of the major hurdles in healthcare. Addressing this problem will require the development of at least 20 new antibiotics by 2060. However, the process of designing new antibiotics is time-consuming. To overcome the spread of drug-resistant microbes and infections, constant evaluation of innovative methods and new molecules is essential. Research is actively exploring alternative strategies, such as combination therapies, new drug delivery systems, and the repurposing of existing drugs. In addition, advancements in genomic and proteomic technologies are aiding in the identification of potential new drug targets and the discovery of new antibiotic compounds. In this review, we explore new sources of natural antibiotics from plants, algae other sources, and propose innovative bioinspired delivery systems for their use as an approach to promoting responsible antibiotic use and mitigate the spread of drug-resistant microbes and infections.

## Introduction

Over time, many bacteria develop ability to tolerate antibiotics, well before humans start mass-producing them as a means to prevent and treat infectious diseases [1, 2]. Currently, there is a great need to find new antibiotics that are effective and safe for society, due to the problems that have arisen related to the decrease and even loss of effectiveness of antibiotics due to an increase in bacterial resistance. While antibiotics are effective in preventing and treating infections in both humans and animals, they are also linked to negative side effects. The emergence of multi-drug resistant and extensively drug-resistant microorganisms poses a significant problem in the treatment of bacterial infections and related co-morbidities [3]. As a result of the concerning emergence and widespread dissemination of antibiotic resistance, coupled with the slow development of novel antibiotics, conventional treatments are progressively becoming less effective [4]. To address this problem, there is a need for new antibiotics and new treatments, but this discovery is a lengthy and time-consuming [5]. Therefore, innovative methods and new molecules are being proposed and evaluated constantly.

Multiple causes contribute to the emergence of antibiotic-resistant bacteria, including: (i) excessive and inappropriate use of antibiotics [6], sometimes as the result of lack of new ones [7], (ii) inadequate infection control strategies [8], (iii) genetic variables, and (iv) environmental factors [9] and (v) natural resistance of the individual that occurs due to natural selective pressure, even with the correct use of the antibiotic.

The highest priority is to look for some alternative that might prevent the development of this drug resistance, and various natural antimicrobial molecules have been identified and explored for their application [10].

Unlike conventional antibiotics, natural bioactive compounds are not chemically modified secondary metabolites extracted from plants, fungi, microbes, or animals, that play an important role in the treatment of diverse pathologies. It is predicted that natural sources harbor a very large number of bioactive molecules that are yet to be discovered, particularly in plants.

One significant benefit of using phytochemicals for antimicrobial treatments is their ability to interact with several classes or subtypes of molecules, known as molecular promiscuity [11]. Traditional antibiotics usually have a specific bacterial molecular target, whereas phytochemicals usually show a multi-targeting capacity, i.e., the same compound showing a significant affinity to several protein targets [11]. The presence of promiscuity or multitarget affinity in bacteria can hamper the development of potential resistance mechanisms [12].

**Fig. 1:**
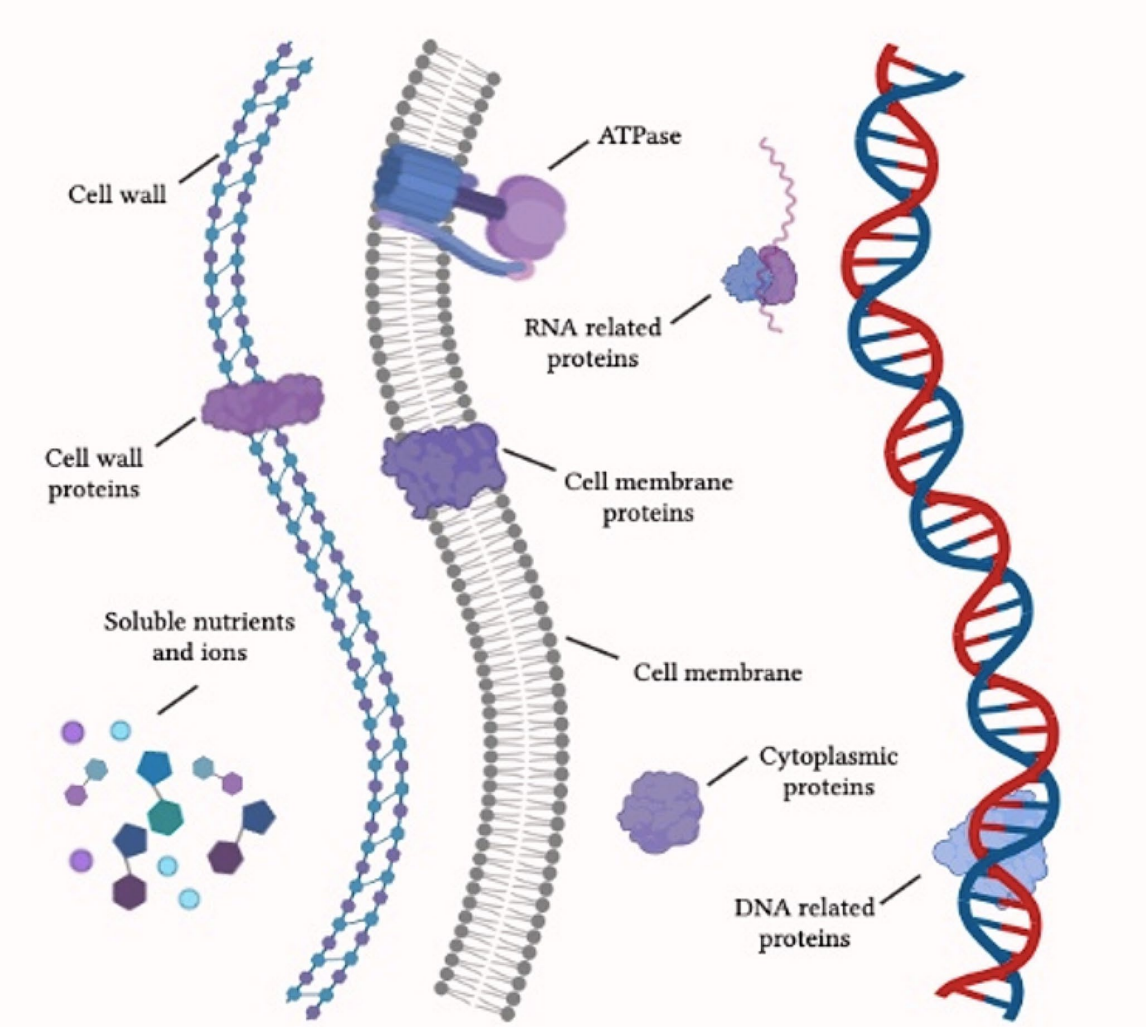
Schematic representation of the main bacterial molecular targets of phytochemicals with antibacterial activity

As shown in Fig. 1, phytochemicals have the potential to target various bacterial structures, such as the cell wall [13] and the components of the cell membrane [14]. They can also interact with proteins that are placed in different parts of the microorganism and have multiple activities [15]. This demonstrates their capability to interfere with nutrition metabolism and motility [16]. Besides their direct pharmacological effects, certain phytochemicals, such as polyphenols, have demonstrated the ability to enhance the susceptibility of antibiotic resistant bacteria. This is achieved by reversing their resistance mechanisms and by increasing their sensitivity to conventional drugs [17, 18].

Since ancient times, plant extracts have been used by humans even when scientific evidence was practically nil and knowledge was limited to trial-and-error screenings [19]. Currently, advancement of modern technology can maximize the use of these phytochemicals and amplify their advantages for human health, as exemplified by nanotechnology [11, 20, 21].

**Fig. 2:**
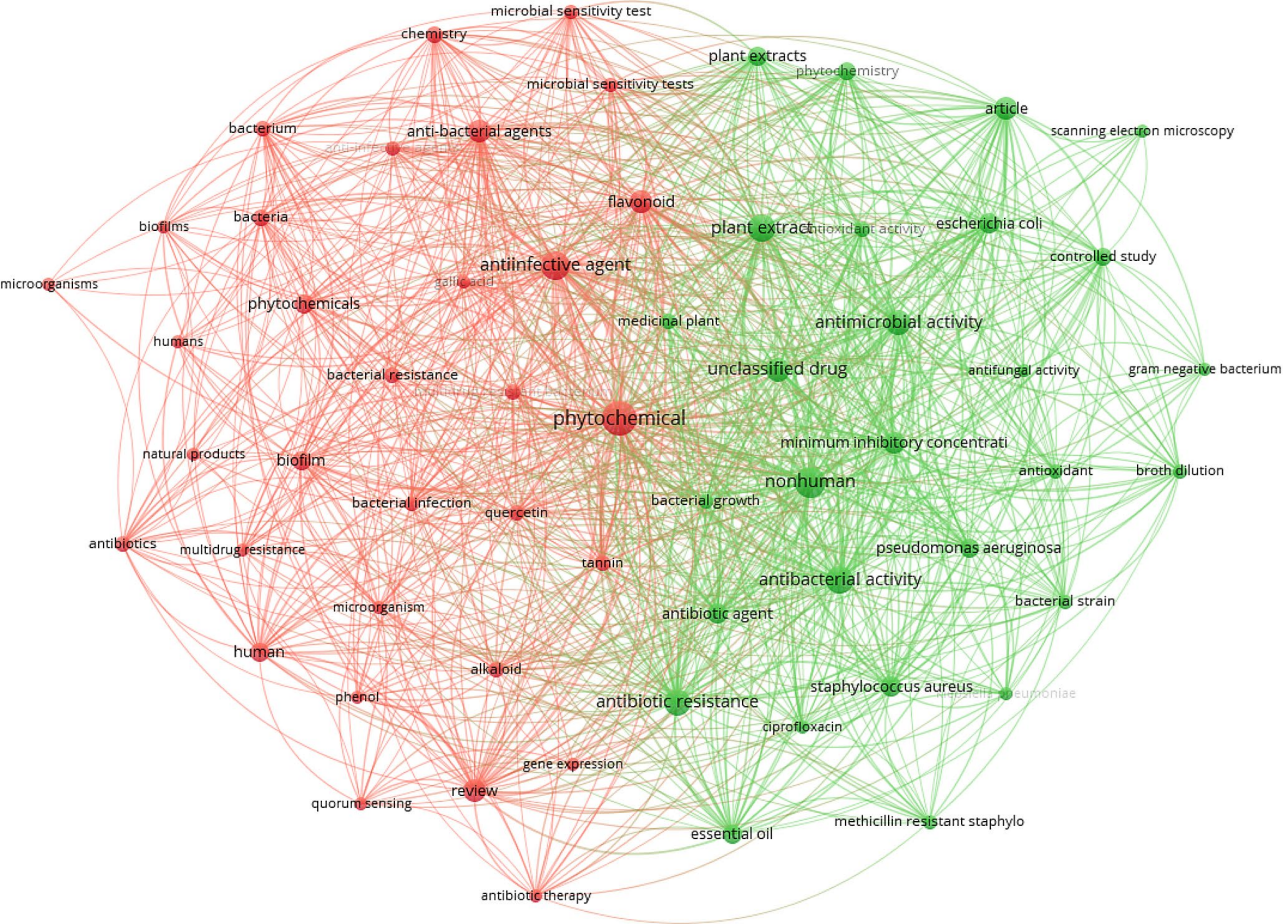
Bibliometric map obtained by VOSviewer software version 1.6.16 (https://www.vosviewer.com), using “phytochemicals” AND “antimicrobial” AND “bacterial resistance” as keywords, recorded from Scopus database limiting the search to documents published from 2021 onwards (recorded on the 3rd May 2024)

In this work, we propose to discuss the use of non-chemically modified natural antibiotics obtained from different sources that show evidence of potential use as alternative anti-microbial compounds to overcome antibiotic-resistant bacteria, and present potential nanotechnological alternatives for a synergistic treatment. To highlight the relevance of this scientific field, a search on Scopus database was conducted, using a combination of terms, i.e., “phytochemicals” and “antimicrobial”, which resulted in a total of 11,926 documents, almost half (5,233) published only since 2021. Refining our search to include the term “bacterial resistance”, a total of 47 papers were retrieved and their abstract and keywords analysed by VOSviewer software to generate the bibliometric map shown in Fig. 2 [22]. Two clusters were generated from this data analysis, the dominating red cluster linking terms such as alkaloid, antibiotic therapy, biofilms, bacterial resistance, microbial sensitive testing and multidrug resistance. The green cluster linked terms such as antibacterial activity, bacterial growth, phytochemicals and minimum inhibitory concentration. The recorded outputs highlight the importance of discussing the potential uses of phytochemicals obtained from e.g., plantae, algae and from other sources, and how they can be exploited to overcome the burdens associated to antibiotic resistance.

## Antimicrobials from plant sources

One of the primary benefits of utilizing phytochemicals for antimicrobial purposes is their ability to interact with several factors or their molecular promiscuity [11]. This multi-target affinity makes it difficult to generate possible resistance mechanisms in bacteria [12].

Medicinal plants are rich in a range of phytochemical compounds, namely alkaloids, coumarins, essential oils, flavonoids, lectin, phenolic, polypeptides, polyacetylenes terpenoids and tannins [23, 24]. These bioactive chemicals have the potential to exhibit bactericidal or bacteriostatic effects on bacteria that are resistant to several drugs. Additionally, these compounds may serve as precursors for the development of antibiotics that can be used to treat such infections [25–27].

The prevalence of antimicrobial resistance is increasing globally, posing a significant challenge to the effective management of numerous infectious diseases [28]. The rise of multidrug-resistance among clinically important bacterial species, and their propensity to form biofilms, is causing a significant public health concern [28]. Treating infections caused by biofilm-forming microorganisms is proving to be a challenging task as eradicating biofilms with conventional antibiotics is becoming increasingly difficult [29]. Several reports have demonstrated that antibiotics frequently fail to eliminate biofilms [30]. Therefore, innovative strategies capable of overcoming the limitations of conventional antibiotics are in demand. Natural chemicals, particularly those derived from plants, have been demonstrating potential uses for antimicrobial treatments [28]. Plant secondary metabolites show antibiofilm properties due to their many modes of action, that are distinct from those of conventional antibiotics. These mechanisms include the suppression of quorum-sensing, motility, adhesion, and reactive oxygen species generation, among others. The combined use of several phytochemicals and antibiotics has demonstrated synergistic or additive effects in the control of biofilms [28].

Biofilms are made of complex microbial communities connected to biological or abiotic surfaces and included in the matrix generated by proteins and polysaccharides [31]. Biofilm creation contributes to the set-up of antibiotic resistance and the generation of persistent cells which are accountable for the uncontrolled persistence of microbial infections [32]. The resistance of biofilms can be attributed to the simultaneous presence of various mechanisms, including limited penetration or deactivation of antimicrobial substances within the biofilm matrix, slow bacterial growth, the existence of persister cells, programmed cell death, and the positive regulation of efflux pumps, among others [33–35]. This inherited characteristic becomes increasingly challenging to eradicate, and is accountable for a serious of problems related to environment, agriculture, industry, and medicine [36].

Different studies describe the antimicrobial activity of *Aloe vera* and its main components, focusing on its antibacterial activity. Methicillin-resistant *Staphylococcus aureus* (MRSA) biofilms growth was reduced by *Aloe vera* aqueous extract [37]. Moreover, these bacteria, along with oral pathogens sampled from patients with periodontal and periapical abscess (e.g., *Clostridium bacilli, Actinobacillus actinomycetemcomitans* and *Streptococcus mutans*) could also be inhibited by *Aloe vera* gel [38]. *Aloe vera* composed-chitosan films were produced to promote antimicrobial effects in wound dressings [39]. Aloe-emodin is a molecule that has been identified as having antibacterial properties against *S. aureus*. It works by preventing the formation of biofilms and the generation of proteins outside the cells [40]. *Aloe vera* extracts also show the ability to counteract the growth of drug-resistant strains of *Pseudomonas aeruginosa* in burned patients with wounds infections [41]. *Aloe vera* gel was successfully applied to reduce the growth and biofilm formation of *P. aeruginosa*, as well as other Gram-negative bacteria (*Escherichia coli* and *Helicobacter pylori*) and fungi (e.g., *Candida albicans*) [42].

Phytochemicals can enhance the effectiveness of antibiotics by acting e.g., as adjuvants [43], to promote their antimicrobial activity. This synergistic effect may result in the reduction the dose of antibiotic needed for the treatment [44], and thus minimizing their adverse effects and ultimately the impact on the environmental [45]. In addition, phytochemicals have the potential to enhance the immune system and the individual’s overall health, thus facilitating defense against infection [46]. An illustration of this phenomenon is the efficacy of utilizing reduced dosages of active compounds from plants for the treatment of infectious disorders. This can be attributed to the presence of a range of active ingredients in plant extracts, which enhances the therapeutic impact in comparison to isolated compounds [47], increasing the plant defense due to their synergistic action. One example of this synergistic effect occurs with tomatoes. When the fruit is attacked by an insect, alkaloids, oxidative enzymes, phenolics and proteinase inhibitors and act synergistically to ingest the threat, with subsequent digestion and metabolism. Likewise, in the species of wild tobacco *Nicotiana attenuata*, inhibitors of nicotine expression and trypsin proteinase act synergistically towards a defensive response against *Spodoptera exigua* (Hub) pest infections [48].

An example of a plant that provides numerous phytochemicals that are beneficial to human health is *Psidium guajava* L., of the plant family Myrtaceae. This plant is a native American shrub that grows in tropical environments worldwide [49]. The many components of the guava tree, such as bark, fruits, leaves, roots and stem, have been utilized in numerous countries for the treatment of stomachache, diabetes, diarrhea, and various other health conditions [50, 51]. The chemical composition of *Psidium guajava* L. includes alkaloids, carbohydrates, flavonoids, phenols, saponins, sterols, tannins and terpenoids [51, 52]. The published literature reports phenolics as the major components of this plant, being capable of interacting with bacterial cell walls, resulting in their rupture and in the leakage of cellular components [53]. This leads to the suppression of a variety of microbial virulence factors (such as toxin production and biofilm formation), and to the inhibition of the synthesis of nucleic acids and the activity of enzymes [54].

Undoubtedly, the leaves of *Psidium guajava* L. are the most analyzed component. These are an abundant reservoir of essential nutrients, including minerals (such as calcium, iron, magnesium, potassium, sodium, sulfur), as well as vitamin B and C [55]. In addition to these compounds, Guava leaves are also rich in essential oils, and the major constituent includes 1,8-cineole and *trans*-caryophyllene [56]. These essential oils display strong cytotoxic and antimicrobial properties against *Bacillus subtilis, E. coli*, *P. aeruginosa*, *Streptococcus faecalis* and *S. aureus* [57].

Guava leaves are recognized for their antibacterial properties because of the presence of various inorganic and organic antioxidants and anti-inflammatory compounds [58].

Our knowledge of the exact mechanism by which antibacterial activity occurs is currently inaccurate, mostly due to the widely diverse structures of phytochemicals, which give rise to numerous potential mechanisms of action. Furthermore, plant extracts consist of an intricate combination of chemicals that can affect their interaction [53]. The mechanism of action is therefore governed by the type of extract or essential oil and the type of microorganism involved in the infection [59].

## Antimicrobials from algae sources

In pursuit of an alternative to conventional antibiotics, many studies are driving their focus on the use of marine sources [60]. Marine organisms are being described as an inexhaustible source of biologically active compounds, producing interesting bioactive molecules useful for treating diverse diseases [61].

Marine algae, a highly abundant oceanic resource, plays a crucial role in the marine ecosystem by serving as a primary food source for marine organisms and offering potentially renewable resources for humans [62]. Marine algae, encompassing both macroalgae and microalgae, inhabit diverse settings and are found in all Earth’s ecosystems [63]. Macroalgae are very suitable for exploiting novel alternative antimicrobials. The global diversity of micro and macroalgae is estimated to be roughly 164,000 species, with around 9,800 of them being marine algae [64].

Algae are photosynthetic organisms that exhibit a wide spectrum of adaptability to unfavorable environmental conditions. They produce vast quantities of secondary metabolites that are effective against a wide range of pathogenic bacteria. Algae from rivers, lakes, and the ocean have been found to possess antibacterial properties against harmful bacteria and fungi [65].

Algae present in the marine environment have a plentiful supply of natural substances that can be used for therapeutic purposes and several other applications. These algae can be found in both prokaryotic and eukaryotic forms, and they inhabit a diverse range of environments, including shallow waters, coastal areas, and backwaters [60, 66]. Many scientific studies have demonstrated that substances extracted from these marine species exhibit antimicrobial properties against both gram-positive and gram-negative bacteria, both in laboratory settings (in vitro) and in living beings (in vivo) [67, 68].

Marine algae can be categorized according to their pigment composition into three types, namely, green (*Chlorophyta*), brown (*Phaeophyta*), and red (*Rhodophyta*) [69, 70]. In recent years, interest on isolating and identifying bioactive chemicals from marine algae, particularly brown macroalgae, has been increasingly growing. A wide range of substances, including specific minerals and phytochemicals, have already been identified for their potential therapeutic properties in the management and prevention of many diseases [71, 72]. Brown seaweeds produce polysaccharides, such as alginates, fucoidans, and laminarins, which have been described for their numerous health advantages. Alginates are known to regulate hunger and have favorable effects on the gastrointestinal tract. They also exhibit antidiabetic and antihypertensive properties [73]. Fucoidans have been associated with the reduction of inflammation, tumor growth inhibition, regulation of the immune system, and with antioxidant and antiviral properties [73]. Laminarins are classified as dietary fibers that can enhance digestive health, and have a crucial role in preventing many disorders, such as colorectal cancer and gastrointestinal inflammation [74, 75].

Because of their numerous biological properties, such as their ability to regulate metabolic diseases, fight cancer, reduce inflammation, and act as an antioxidant, polyphenols are regarded as one of the most promising bioactive substances among other phytochemicals [76]. Marine algae can provide a range of polyphenols, including anthraquinones, benzoic acid, catechins, cinnamic acid, flavonoids, isoflavones, lignans, phenolic acids, phlorotannins and quercetin [67, 77].

Powerful poultry-associated foodborne pathogens responsible for salmonellosis are Salmonella species, in particular *S. Typhimurium S. Enteritidis*. New drug resistant Salmonella strains have also been described attributed to the excessive use of antibiotics [78]. Methanolic extract of *Padina gymnospora* (brown algae) displayed large inhibition zones (27 mm) against *S. typhimurium* [67], while red seaweeds aqueous extracts, *Chondrus crispus* and *Sarcodiotheca gaudichaudii*, potentially inhibit the growth of *S. Enteritidis* at the minimum inhibitory concentration (MIC) value of 15 µg/mL [78].

Extracts from *Padina* and *Ulva* sp. revealed antibacterial activity against *Bacillus cereus, Listeria monocytogenes* and *S. aureus* at concentrations below 500 µg/mL [79]. Srikong et al. (2017) [80] showed that crude extracts derived from the marine alga *Ulva intestinalis* exhibit strong antibacterial properties against *B. cereus, S. aureus, Enterococcus faecalis* and *L. monocytogenes*. The registered MIC and MBC values ranged from 256 to 512 µg/mL.

Marine algae exhibit antimicrobial activity against both bacterial cells and the production of biofilms. A study conducted in 2019, by the Waterford Institute of Technology in Ireland, examined the antibiofilm properties of extracts derived from two types of brown algae, *Fucus serratus* and *Fucus vesiculosus*, against MRSA. The results showed that these extracts were able to completely inhibit bacterial growth and reduce biofilm formation by over 80%. The MIC and MBC values for these extracts were found to be 3.125 and 25 mg/mL, respectively [81].

## Antimicrobials from other sources

The use of natural products as a form of complementary treatment is currently receiving more attention. Propolis, a natural product, has gained attention for its several advantageous properties, making it a valuable option [82–84]. Propolis is a naturally occurring resinous substance that is gathered by several species of bees from diverse plant sources.

Bees collect resins and beeswax from many plant sources, including buds, exudates, flowers, gums, leaf resins, and mucilage found near their hive. They then enhance these substances with their β-glucosidase enzymatic saliva [85–88].

Bee products, particularly propolis, are extensively used in both traditional and alternative medicine. Propolis has been extensively mentioned in ancient medicine due to its manifold health advantages [89].

More than 500 types of compounds are found in propolis, including amino acids, aromatic acids, coumarins, essential oils, esters, flavonoids, minerals, polyphenols, sugars, terpenes, terpenoids, steroids, and vitamins have been identified in propolis [85, 90–92]. Its vitamins (A, B complexes, C and E), and important minerals, such as aluminum, calcium, copper, iron, magnesium, potassium, sodium and zinc [93, 94], play important roles in propolis biological activity [95]. Various factors can modify the composition of propolis. Changes in texture, fragrance, and color of honey occurs as a result of the specific plant parts collected by bees and the bee species involved [92]. Moreover, the antibacterial effects differ significantly across the bacterial strains and is dependent upon the specific propolis sample employed [96]. The medicinal benefits of propolis are mostly ascribed to its volatile components [97, 98], i.e., flavonoids and phenolic compounds. These chemicals are widely recognized for their antioxidant and antibacterial characteristics [99, 100]. Flavonoids present in propolis function by scavenging free radicals and by stimulating antioxidant enzymes. As a result, they protect the cell membrane from oxidative stress, including cellular aging, and against cardiovascular and brain disorders [101]. Many scientific studies describe the antibacterial activity of propolis and its derivatives against several *Bacillus, Enterococcus* and *Streptococcus* species, as well as against *E. coli*, *S. aureus*, *Salmonella typhi* and Pseudomonas [88, 102, 103]. The anticancer and cytotoxicity properties of propolis mostly stem from chrysin, a plant flavone derived from *Passiflora caerulea* leaves. Several reports describe that this flavone possesses antibacterial effects due to its capacity to disrupt the structural integrity of the microbial cell wall and cell membrane [104, 105]. Furthermore, its ability to combat pathogenic yeasts, such as Candida sp., through its antifungal activities, presents a potential alternative for treating [106]. Propolis has been demonstrated to possess antiviral properties, as shown by Yildirim et al. (2016) [107]. Specifically, it has been shown to reduce the reproduction of the *Herpes simplex* virus in vitro, which is responsible for causing orofacial and genital infections. Red propolis hydroalcoholic extract was formulated in mucoadhesive polymeric membranes developed wound healing. These membranes, composed of collage, chitosan, polyethylene glycol and bearing 0.5% (m/V) of red propolis showed MIC values as low as 7.8 and 1.9 µg//mL for *S. aureus* and *P. aeruginosa*, respectively [108].

## Bioinspired delivery systems

New strategies proposed to overcome antibiotic resistance against bacterial infections include the development of nanotechnology-based delivery systems for selected phytochemicals with antimicrobial properties. Besides promoting targeted drug delivery and enhanced bioavailability [109–111], these nanomaterials also offer advantages related to mechanical, physicochemical, biopharmaceutical and modified-release properties [11, 20, 21].

Nanomaterials, that include nanoparticles (NPs) and nanofibers (NFs), can deal with limitations related to traditional strategies [112], offering the opportunity to be surface-tailored to show site-specific targeting approaches and reduce the risk of systemic drug exposure [113]. Besides the advantages related to their size and high surface-to-volume ratio, shape, composition and morphological properties, their mechanical, biological, and physicochemical properties can also be adjusted to meet the required needs [114]. The raw materials composing NPs should be biocompatible [115] and can be selected to be improve the biopharmaceutical properties (i.e., permeability and solubility) of antimicrobial drugs and increase their bioavailability for a certain administration route of selected phytochemicals [116]. Additionally, these characteristics can enhance the biopharmaceutical properties of the end products, particularly focusing on molecules with low bioavailability [117].

**Fig. 3:**
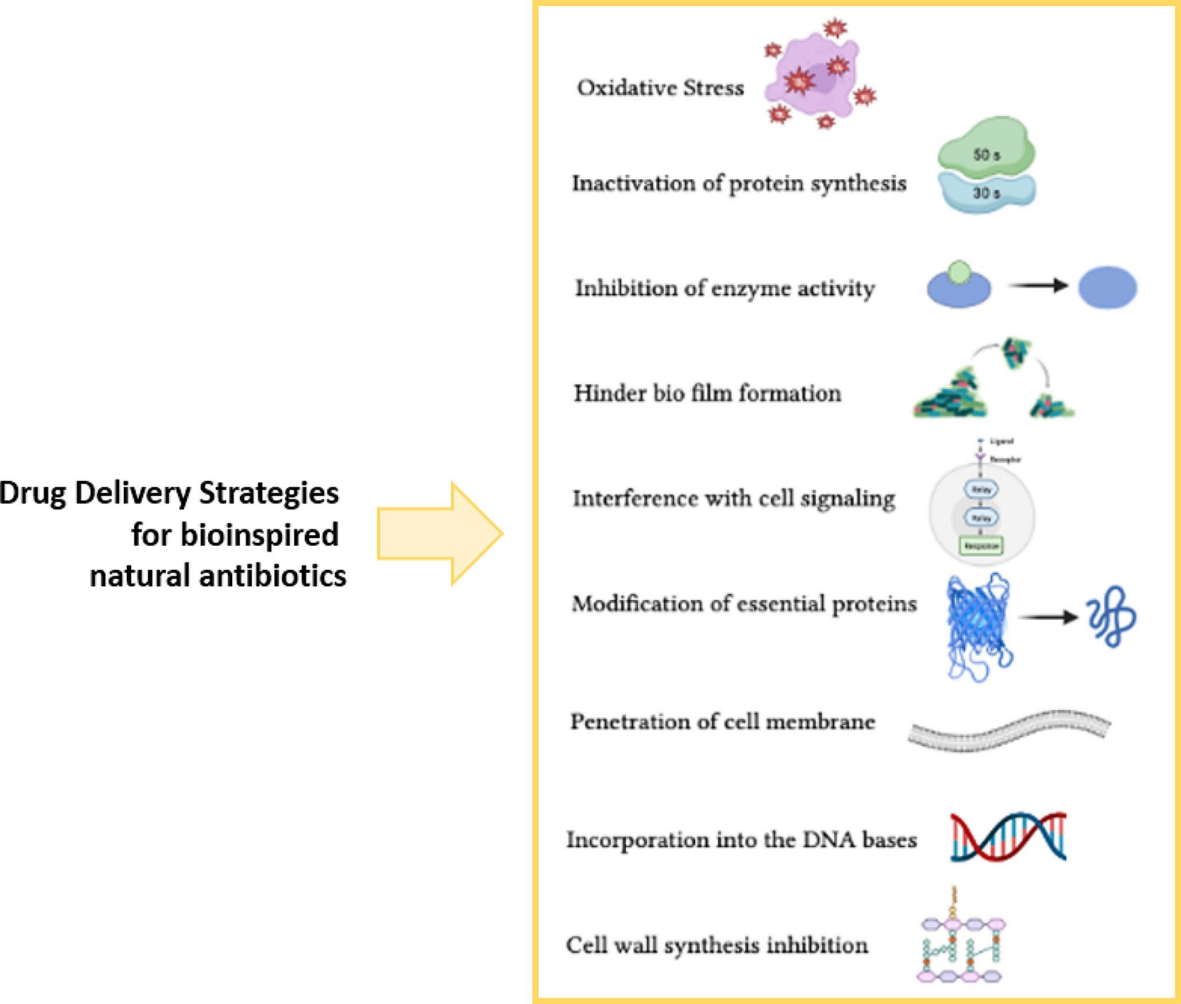
Examples of delivery strategies that can be applied for bioinspired natural antibiotics

The potential to reduce the administered dose of drug [118], the enhanced half-life of loaded drug to be kept longer in the circulatory system for longer periods [119] and customization for precision medicine [120] are additional advantages attributed to NPs. Figure 3 depicts examples of drug delivery strategies that can be applied for bioinspired natural antibiotics. Table 1 summarizes recent applications of the use of drug delivery systems for these bioactives.

**Table 1:** Examples of polymeric nanoparticles loaded with phytochemicals against antibiotic resistant bacteria (reproduced after Díaz-Puertas et al. (2023) [11], under the terms and conditions of the Creative Commons Attribution (CC BY) license

Type of Polymer	Phytochemical	Synthesis	Mean Diameter (nm)*	Antibacterial Activity*	References
Chitosan	Cardamom essential oil	Ionic gelation	50–100	Growth control for 2 days (*E. coli*, MRSA, ESBL)	[121]
Chitosan	*Eucalyptus globulus* leaf extract	Green synthesis	7–10	Zone of inhibition of 12–30 (multi drug- resistant *Acinetobacter baumannii*)	[122]
Chitosan / hydroxypropyl methylcellulose	*Schinopsis brasiliensis* leaf extract /Ceftriaxone	Polyelectrolytic complexation (coacervation)	150–500	MIC of 15 µg/mL (KPC, ESBL)	[123]
Polylactic acid / polyvinyl alcohol	*Pistacia lentiscus* var. *chia* essential oil	Solvent evaporation	240–665	MIC higher than 3.4 mg/mL (drug-resistant *Bacillus subtilis* sub. *Spizizenii*)	[124]

Captions: ESBL, extended spectrum beta-lactamases; KPC, *Klebsiella pneumoniae* carbapenemase, MIC, minimum inhibitory concentration; MRSA, methicillin-resistant *S. aureus;* *Mean values or a range of values are indicated in studies employing various conditions or concentrations

Metal nanoparticles (MNPs) composed of elemental metals (such as Au, Ag, Cu, Fe, Pt, Pd, Ti, or Zn) or their corresponding compounds (such as CuO, Fe3O4, TiO2, ZnO). These particles have sizes ranging from 1 to 100 nanometers. MNPs exhibit distinct physical and chemical characteristics that deviate from those of larger metal structures, owing to the impact of their reduced dimensions and elevated surface area-to-volume ratio [125]. These nanomaterials are being currently exploited in the fields of biomedical sciences and engineering because of their unique characteristics, including exceptional mechanical and thermal stability, large surface area, and remarkable optical and magnetic properties [126].

The nano-scale dimensions, morphology, surface charges, surface functionalization, and drug delivery capabilities of MNPs, which also possess antimicrobial activity [127], serve as a synergistic approach against multidrug-resistance bacterial infections [128, 129]. The main function of these MNPs is to reduce the capacity resistance of bacteria. This is achieved by disrupting membrane potential and bacterial cells integrity, inhibiting biofilms, promoting the formation of reactive oxygen species (ROS), boosting the immune responses of the host, and inhibiting RNA and protein synthesis through the induction intracellular processes [127, 129]. Moreover, the combination of conventional antibiotics with MNPs demonstrates a synergistic impact in decreasing drug-resistant bacterial infections [130, 131]. Table 2 summarizes some examples of green-synthesized MNPs using phytochemicals against antibiotic resistant bacteria.

**Table 2:** Examples of green-synthesized metal nanoparticles using phytochemicals against antibiotic resistant bacteria (reproduced after Díaz-Puertas et al. (2023) [11], under the terms and conditions of the Creative Commons Attribution (CC BY) license

Metal Nanoparticles	Phytochemical	Mean diameter (nm)	MIC (µg/mL) *	References
AgNPs	*Aloe vera* extract	38.9	4.9–9.8 (KPC)	[132]
	*Cinnamomum tamala* LE	10–12	12.5 (MDR *E. coli*), 10 (MDR *K. pneumoniae*, 12.5 (MDR *S. aureus*)	[133]
	*Cotyledon orbiculate* LE	106–137	40 (MRSA)	[134]
	*Flavopunctelia flaventior* powder	69	0.156 (MRSA), 0.078 (VRE), 0.019 (MDR *Pseudomonas aeruginosa)*, 0.078 (MDR *E. coli*)	[135]
	*Mespilus germanica* LE	17.6	6.25–100 (MDR *K. pneumoniae*)	[136]
	*Momordica charantia* extract	9.6–16.4	4 (CR *A. baumannii)*, 4 (IR *A. baumannii*)	[137]
	*Periploca hydaspidis* extract	68.6–114.2	10 (MDR *K. pneumoniae)*, 10–20 (MDR *S. aureus*), 10 (MDR *E. coli)*, 5 (MRSA)	[138]
	*Stenocereus queretaroensis* PE	60–200	0.313 (MRSA)	[139]
	*Syzygium cumini* LE	10–15	8 (MRSA), 20 (VRSA)	[140]
	*Xanthoria parietina* powder	145	0.078 (MRSA), 0.156 (VRE), 0.039 (MDR *P. aeruginosa)*, 0.156 (MDR *E. coli*)	[135]
AuNPs	*Anabaena spiroides* extract	80	25 (MDR *Klebsiella oxytoca*), 30 (MDR *Steptococcus pyogenes*), 20 (MRSA)	[141]
	*Punica granatum* extract	39.4	15.6 (MRSA)	[142]
CuNPs	*Syzygium cumini* LE	30–31	14 (MRSA), 16 (VRSA)	[140]
CuONPs	*Camellia sinensis* extract	61	125 (CREC), 125 (CRKP), 30 (MRSA)	[143]
	*Prunus africana* BE	68	125 (CREC), 125 (CRKP), 30 (MRSA)	[143]
FeNPs	*Syzygium cumini* LE	40–46	11 (MRSA), 13 (VRSA)	[140]
PdNPs	*Padina boryana* extract	8.7	125 (MDR *S. aureus*), 62.5 (MDR *E. fergusonii*), 62.5 (MDR *A. pittii*), 62.5 (MDR *P. aeruginosa*), 62.5 (MDR *A. enteropelogenes*), 125 (MDR *P. mirabilis*)	[144]
TeNPs	*Aloe vera* extract	20–60	11.61 (MRSA), 3.53 (MDR *E. coli*)	[145]
ZnONPs	*Acacia nilotica* extract	94	0.45 (KPC)	[146]

Captions: BE, bark extract; CR, colistin-resistant; CREC, carbapenem-resistant *E. coli*; CRKP, carbapenem-resistant *K. pneumoniae*; FE, flower extract; IR, imipenem-resistant; KPC, *Klebsiella pneumoniae* carbapenemase; LE, leaf extract; MDR, multi-drug-resistant; MREC, methicillin-resistant *E. coli*; MRSA, methicillin-resistant *S. aureus*; NPs, nanoparticles; PE, peel extract; VRE, vancomycin-resistant *Enterococci*; VRSA, vancomycin-resistant *S. aureus*. * Mean values or a range of values are indicated in studies employing various conditions or concentrations

## Conclusions

The use of nanotechnology combined with phytochemical compounds from natural sources i.e., non-chemically modified antimicrobials obtained from different sources (e.g., plant, algae and others) offers several advantages to overcome the serious threat of antibiotic-resistant bacteria. Nanomaterials can address limitations associated with traditional approaches and provide beneficial morphologies and surface features against bacterial infections. The characteristics of nanomaterials, such as size, shape, composition, and surface properties, can be adjusted to match the specific requirements of antimicrobial therapy. For example, polymeric matrices can result in small size and high surface-to-volume ratio, enhancing the permeability and solubility of loaded phytochemicals. This property is particularly advantageous for drug delivery, as it can improve the bioavailability of phytochemicals and enhance their antimicrobial effects with less drug systemic exposure. Polymeric nanoparticles offer several desirable properties for the encapsulation of antimicrobial drugs of natural origin. They enable controlled release of the loaded antimicrobial agents, allowing for sustained, prolonged or extended activity against bacteria. Nanoparticles also offer the possibility of targeted delivery, where the encapsulated drugs can be directed specifically to the site of infection, thereby minimizing systemic exposure and potential side effects minimizing the risk of toxicity. Properties, such as biocompatibility and tolerability, are instrumental for their safe use in biomedical applications. Nanoparticles can circulate in the bloodstream for longer periods, providing an extended duration of action. This feature is particularly beneficial for chronic or persistent bacterial infections. The possibility to customize their properties, i.e., tailoring nanoparticles to specifically meet the therapeutic needs, allows the development of precision medicine strategies. The same applies for metal nanoparticles, which became popular as they can be produced from natural sources using different types of phytochemicals. These nanoparticles naturally exhibit antibacterial properties that can further be exploited as novel types of antibiotics. Overall, the use of nanotechnology in combination with antibacterial phytochemicals holds promise to overcome the challenges posed by antibiotic-resistant bacteria. By leveraging the unique properties of nanomaterials, it is possible to enhance the antibacterial capacity against resistant strains and develop more effective strategies for treating bacterial infections. The need to find new antibacterial agents that are effective against antibiotic resistant bacteria is in demand and nanotechnology is showing significant advancements for this purpose.

## Data Availability

This work does not contain authors own data.
